# Naupliar and Metanaupliar Development of *Thysanoessa raschii* (Malacostraca, Euphausiacea) from Godthåbsfjord, Greenland, with a Reinstatement of the Ancestral Status of the Free-Living Nauplius in Malacostracan Evolution

**DOI:** 10.1371/journal.pone.0141955

**Published:** 2015-12-18

**Authors:** Hasna Akther, Mette Dalgaard Agersted, Jørgen Olesen

**Affiliations:** 1 Natural History Museum of Denmark, University of Copenhagen, Copenhagen, Denmark; 2 National Institute of Aquatic Resources, Section for Oceanography and Climate, Technical University of Denmark, Charlottenlund, Denmark; National Taiwan Ocean University, TAIWAN

## Abstract

The presence of a characteristic crustacean larval type, the nauplius, in many crustacean taxa has often been considered one of the few uniting characters of the Crustacea. Within Malacostraca, the largest crustacean group, nauplii are only present in two taxa, Euphauciacea (krill) and Decapoda Dendrobranchiata. The presence of nauplii in these two taxa has traditionally been considered a retained primitive characteristic, but free-living nauplii have also been suggested to have reappeared a couple of times from direct developing ancestors during malacostracan evolution. Based on a re-study of *Thysanoessa raschii* (Euphausiacea) using preserved material collected in Greenland, we readdress this important controversy in crustacean evolution, and, in the process, redescribe the naupliar and metanaupliar development of *T*. *raschii*. In contrast to most previous studies of euphausiid development, we recognize three (not two) naupliar (= ortho-naupliar) stages (N1-N3) followed by a metanauplius (MN). While there are many morphological changes between nauplius 1 and 2 (e.g., appearance of long caudal setae), the changes between nauplius 2 and 3 are few but distinct. They involve the size of some caudal spines (largest in N3) and the setation of the antennal endopod (an extra seta in N3). A wider comparison between free-living nauplii of both Malacostraca and non-Malacostraca revealed similarities between nauplii in many taxa both at the general level (e.g., the gradual development and number of appendages) and at the more detailed level (e.g., unclear segmentation of naupliar appendages, caudal setation, presence of frontal filaments). We recognize these similarities as homologies and therefore suggest that free-living nauplii were part of the ancestral malacostracan type of development. The derived morphology (e.g., lack of feeding structures, no fully formed gut, high content of yolk) of both euphausiid and dendrobranchiate nauplii is evidently related to their non-feeding (lecithotrophic) status.

## Introduction

It has traditionally been difficult to identify characters shared by all or at least most of the morphologically diverse Crustacea. This may not be surprising considering the great age of Crustacea (e.g., [1]). Adding to the difficulties of finding commonalities for Crustacea is the growing evidence of its paraphyly with respect to Hexapoda (insects and allies) [2, 3]. One of the classical candidates for a uniting set of characters for Crustacea is the ‘nauplius larvae’, a characteristic larval type with only three pairs of appendages: first antennae, second antennae, and mandibles (see reviews in [4, 5]). A naupliar developmental phase is present in practically all major crustacean taxa [6]. Despite the likely paraphyly of Crustacea, shared larval types such as the nauplius still potentially holds important evolutionary information, e.g., for phylogeny, or at least for explaining the evolution of the Crustacea, in which heterochrony (= evolution caused by developmental changes in timing of events) has played an important role.

The study of crustacean larvae is an old discipline in the attempt of elucidating the evolution of Crustacea [7] and has in recent years received renewed attention. Developmental stages of many crustacean groups have been examined in recent years offering new details [8–17]. Adding to this is the wealth of data on the development of Crustacea (and related groups) from the Cambrian, many of which go through an early phase of naupliar or naupliar-like stages [18–20]. It is now firmly established that the study of early larval development of Crustacea and other arthropods is indispensable if a full understanding of the evolution of these taxa is to be achieved. Concerning the crustacean nauplius stage, it has recently been discovered that also Remipedia go through an early developmental phase with naupliar-like stages [21, 22]. Hence, a nauplius type of larvae is indeed widespread within the Crustacea, and occurs in practically all major taxa (see details in [6]).

Also within Malacostraca, which holds the largest proportion of crustacean diversity (e.g., Decapoda and Peracarida), a couple of taxa are well-known to start their development with a phase of free-living nauplii. These are the euphausids (krill) and dendrobranchiate decapods, both of which are pelagic taxa with a general ‘caridoid facies’-like (shrimp-like) appearance. The classical notion is that the presence of a naupliar phase in the early development of these two taxa is ancestral in Malacostraca and has been either lost or modified in several malacostracan subtaxa [23–25]. An alternative view is that the presence of free nauplii has reappeared secondarily during malacostracan evolution from direct-developing ancestors [26, 27].

**Fig 1:**
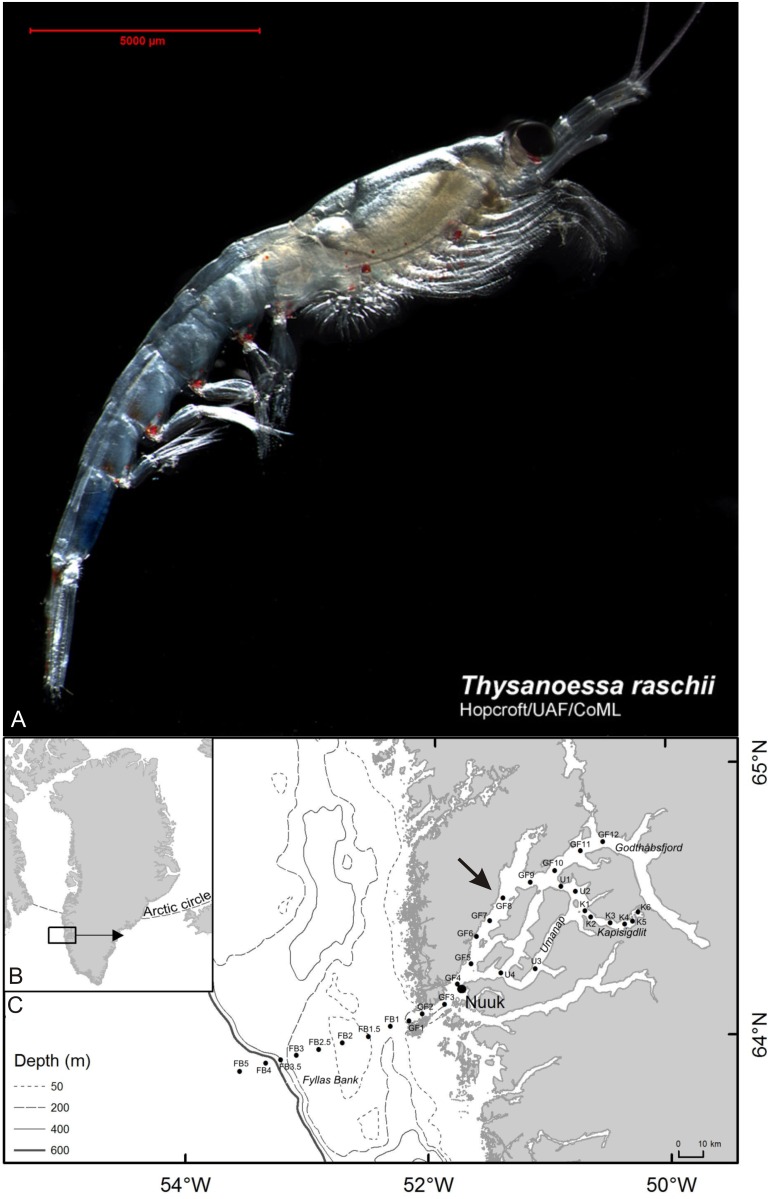
*Thysanoessa raschii* (Euphausiacea), adult morphology and map of sampling sites. (A) Adult morphology of *Thysanoessa raschii* (Euphausiacea) (photo by permission from Russel Hopcroft). (B, C) Map of Godthåbsfjord, SW Greenland, with sampling stations indicated by black dots and station numbers. The larvae examined in this study originated from station GF8 (arrow on C).

Here we use scanning electron microscopy to describe the naupliar and metanaupliar phase of *Thysanoessa raschii* (Fig 1), a broadcast-spawning species of Euphausiacea. Euphausiaceans go through a number of phases during their development: a nauplius (= ortho-nauplius) phase, a metanauplius ‘phase’, a calyptopis phase, and a furcilia phase, each of which may contain more than one larval stage, but with a relatively constant morphology within each phase [28]. In the present study we only focus on nauplii and metanauplii. We recognize three naupliar stages, in contrast to earlier studies, and describe/re-describe some previously unknown or poorly understood aspects of the external morphology of early euphausiid development. Moreover, we compare euphausiid naupliar morphology with that of other crustacean nauplii, and use this to discuss the evolutionary status of malacostracan nauplii.

## Materials and Methods

### Collecting of material and morphological studies

The larval material of *Thysanoessa raschii* (Euphauciacea) (Fig 1A) used for this study was collected in the inner part of Godthåbsfjord, Greenland (sampling site indicated on Fig 1B) (see also [29]). The sampling was part of a project (BOFYGO—Biological Oceanography of Fyllas Bank-Godthåbsfjord)) coordinated by Professor Torkel Gissel Nielsen (DTU Aqua), which took place from 7 to 22 June 2010 from RV “Dana” (National Institute for Aquatic Resources, Denmark). No permit for collecting zooplankton in Greenland is required. The field studies did not involve endangered or protected species. Background information on the oceanography and the composition and distribution of plankton are reported in [29–31]. Plankton was collected on 15 June 2010 at station GF8 (Fig 1B) by oblique hauls to 110 m depth with a Bongo net (300 μm and 500 μm mesh size) with non-filtering cod-ends. Only plankton from the 300 μm net was used in the present study. It was preserved in buffered formalin (4% final conc.) immediately after collection. Krill larvae were sorted from the sample and separated roughly into different developmental categories: nauplius, metanauplius, calyptopis, and furcilia (latter two not included in the present study, see e.g. [32]).

The first three stages have been termed ‘nauplii’ thereby following the literature on early development of Euphauciacea. Some treatments dealing with general aspects of crustacean development use the term ‘ortho-nauplius’ for those nauplii with only three pairs of functional appendages and with no anlage to a fourth pair(maxillae 1) (e.g., [4, 6, 33].

**Fig 2:**
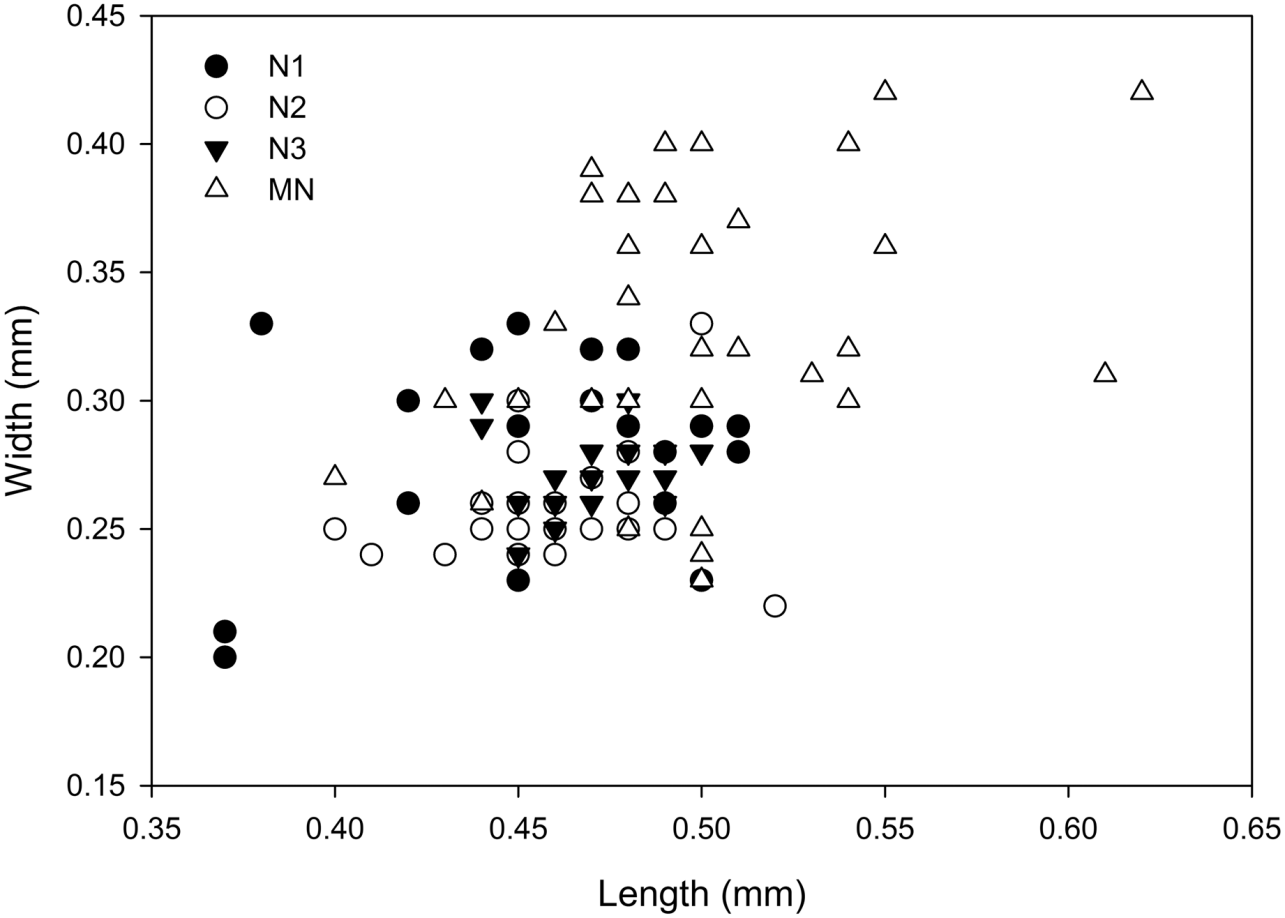
Size of nauplii 1–3 (N1-N3) and metanauplius (MN) of *Thysanoessa raschii* (Euphausiacea). Illustrated as width plotted against length (in millimeters) based on data shown in Table 1.

**Fig 3:**
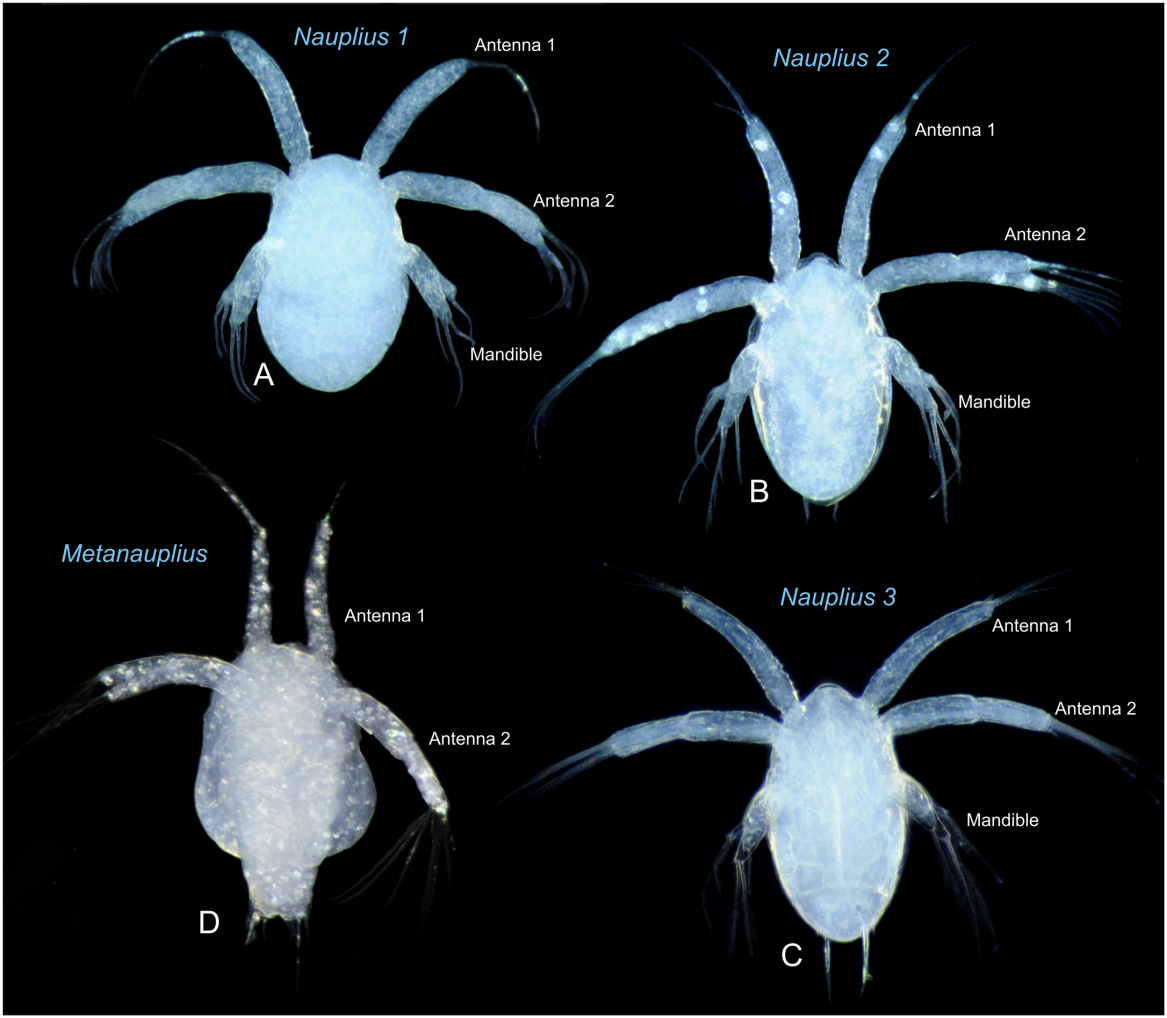
Nauplii 1–3 and metanauplius of *Thysanoessa raschii* (Euphausiacea), light microscopy. (A) Nauplius 1. (B) Nauplius 2. (C) Nauplius 3. (D) Metanauplius. The images are not to the same scale. See Table 1 and Fig 2 for size variation of the larvae.

About 400 nauplii (= ortho-nauplii) and metanauplii were selected for various types of morphological analysis. Standard measurements of body size (length versus width) (Fig 2) and general overview photographing (Fig 3) took place under a dissecting microscope Olympus SZX10 with a Nikon D700 camera fitted via an LM adapter. The Nikon camera was tethered to a PC with the program ControlMyNikon v. 4.3. Some morphological aspects were examined and photographed in more detail using a compound microscope Nikon Microphot-FX fitted with an Olympus DP73 camera. For both types of microscopes, a larger depth of field was obtained by combining several photographs taken at different focal points with the software Zerene Stacker v. 1.04. Most morphological information was obtained from SEM (scanning electron microscope) studies of selected specimens. The SEM used was a JEOL JSM-6335F (with a field emission gun). The material for SEM was dehydrated in an ethanol series, critical point dried, mounted and coated with platinum/palladium following standard procedures (e.g., [34]). In the initial phase of the work with the SEM, many problems were encountered regarding cuticle shrinkage and even more critical collapse of tissue, rendering the results largely useless. These problems were in part overcome by post-fixing some specimens in 1% osmium tetroxide. To obtain maximum resolution at the SEM, some of the whole view illustrations were combined (stitched) from several images taken at high magnification. The images were processed and photo plates were made in standard graphical software such as CorelDraw X7 and various Adobe programs. The SEM material on which the work is based is stored in the collection of the Natural History Museum of Denmark and can be made available after contacting the collection unit at this museum (e.g., the Curator of Crustacea, Jørgen Olesen, jolesen@snm.ku.dk). All SEM material is stored under registration numbers ZMUC-CRU-4797 to ZMUC-CRU-4799.

In the present study the analyzed krill larvae originated from station GF8 (Fig 1B), where adult krill abundance was dominated by *Thysanoessa raschii*, followed by *Meganyctiphanes norvegica* and *T*. *inermis* [29]. Adult individuals from st. GF1 (mouth of Godthåbsfjord) and st. GF11 and GF12 (inner part of Godthåbsfjord) were analyzed with regard to maturity. About half of the mature females of *T*. *raschii* were fertilized both at the mouth of the fjord (st. GF1) and in the inner part (st. GF11-GF12), whereas fertilized females of *T*. *inermis* were only found at the mouth of the fjord (GF1) [29]. Juveniles and fertilized females of *M*. *norvegica* were not present in the fjord, which suggests that individuals of this species are seeded from the offshore population by advection and that *M*. *norvegica* does not reproduce this far north [29]. Additionally, the fjord’s side branch Kapisigdlit (Fig 1B) was also dominated by *T*. *raschii* and all of the mature females there were fertilized. Conversely, *T*. *inermis* were not so abundant and its females were not fertilized [29]. Therefore, based on the fact that only fertilized females of *T*. *raschii* were present in the inner part of the fjord, and on the fact that *T*. *raschii* dominated at st. GF8, we assume that all of the larvae analyzed in the present study belong to *T*. *raschii*.

### Statistical analysis

As length and width data were not normally distributed, the data were log transformed prior to analysis. By one-way ANOVA it was tested whether there was any difference in either individual length or width between the four different larval stages [35]. Afterwards, we performed pairwise comparisons using the Tukey multiple comparisons of means posthoc test.

## Results

### Measurements of larvae

Based on morphological criteria it was possible to distinguish clearly between three naupliar stages (in the following termed N1-N3), and one metanauplius stage (MN). In the initial phase of the work, the larval specimens were divided based on spine development of the caudal region, a division later confirmed by more detailed studies of development of limb setation (see below). Many (25–45) specimens of each stage were selected and measured (length *versus* width; mm) (Table 1) under the dissection microscope and plotted in a diagram in order to reveal any grouping of the specimens based on size alone (Fig 2). Length was measured from anterior to posterior without including the caudal spines. The width was measured at the widest place of the body. This yielded no clear-cut grouping of the specimens into categories based on size. The average lengths and width of N1-3 were about the same (450–470 μm and 250–270 μm, respectively). Most size variation (both length and width) (expressed as standard deviation, Table 1) was seen among N1 specimens, less among N2 specimens, and least among N3 specimens. There was a wide range in size among the measured MN specimens, and this stage included the largest larval specimens found in this study (Fig 2). There was a significant difference in length (ANOVA, F_3, 122_ = 14.04; p<0.001) and width (ANOVA, F_3, 122_ = 27.55; p<0.001) between stages. However, a Tukey posthoc test revealed no significant difference between N1, N2 and N3, whereas all three nauplii stages were significantly different from the metanauplius stage (all p-values <0.01).

**Table 1:** Sizes of nauplii (N1-3) and metanauplii (MN) based on measurements of body length and width (mm) (see text).

	Nauplius 1 (*n* = 28)		Nauplius 2 (*n* = 28)		Nauplius 3 (*n* = 25)		Metanauplius (*n* = 45)	
	Length (mm)	Width (mm)	Length (mm)	Width (mm)	Length (mm)	Width (mm)	Length (mm)	Width (mm)
***Mean size***	***0*.*45***	***0*.*27***	***0*.*46***	***0*.*26***	***0*.*47***	***0*.*27***	***0*.*50***	***0*.*33***
**Variation**	0.37–0.51	0.20–0.33	0.40–0.52	0.22–0.32	0.44–0.50	0.24–0.30	0.40–0.66	0.23–0.49
**Standard deviation**	0.040	0.035	0.027	0.021	0.016	0.015	0.047	0.055

### Description of nauplii 1–3 and metanauplius

**Fig 4:**
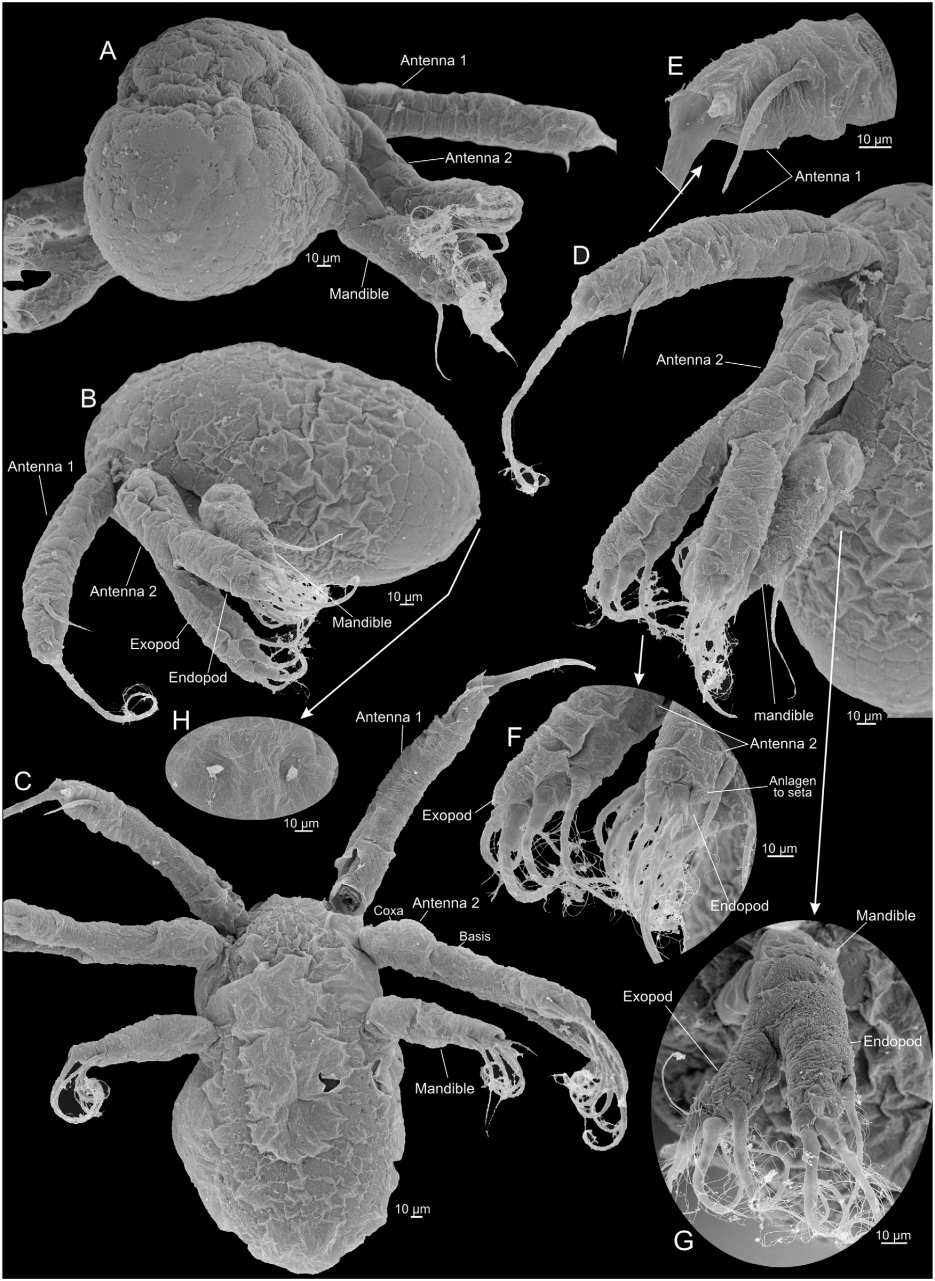
Nauplius 1 of *Thysanoessa raschii* (Euphausiacea), scanning electron microscopy (SEM). (A) Posterior view of whole specimen. (B) Lateral view of whole specimen. (C) Ventral view of whole specimen. (D) Ventral view of right side of whole specimen. (E) Antenna 1, distal setation. (F) Antenna 2, distal setation of endopod and exopod. (G) Mandible, right side, endopod and exopod. (H) Anlagen to caudal setae.

**Fig 5:**
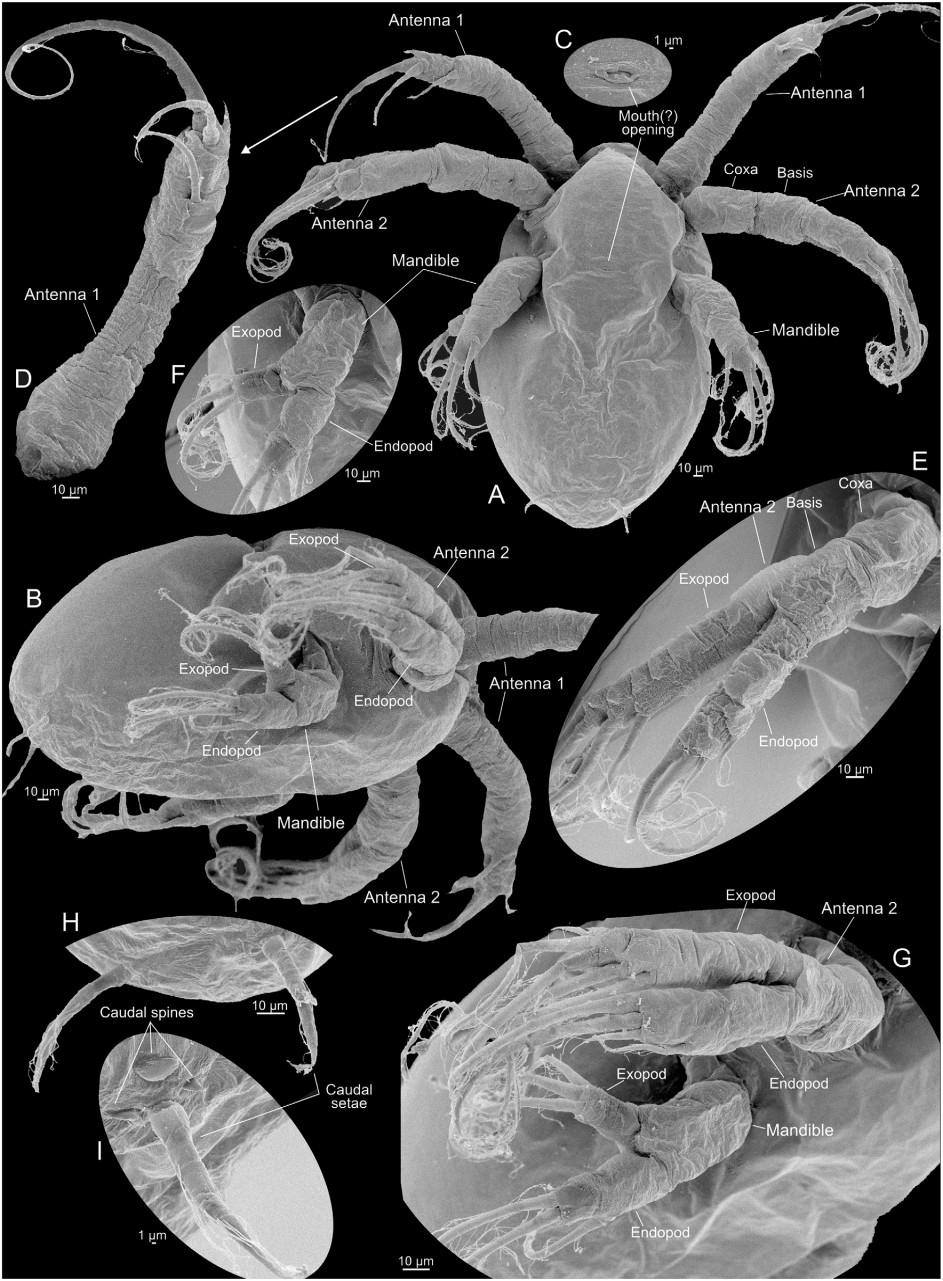
Nauplius 2 of *Thysanoessa raschii* (Euphausiacea), scanning electron microscopy (SEM). (A) Ventral view of whole specimen. (B) Lateral view of whole specimen. (C) Incipient mouth opening (?). (D) Antenna 1, right side. (E) Antenna 2, right side, anterior view. (F) Mandible, right side, anterior view. (G) Antenna 2 and mandible, right side. (H) Caudal setae, ventral view. (I) Caudal seta, left side, posterior view.

**Fig 6:**
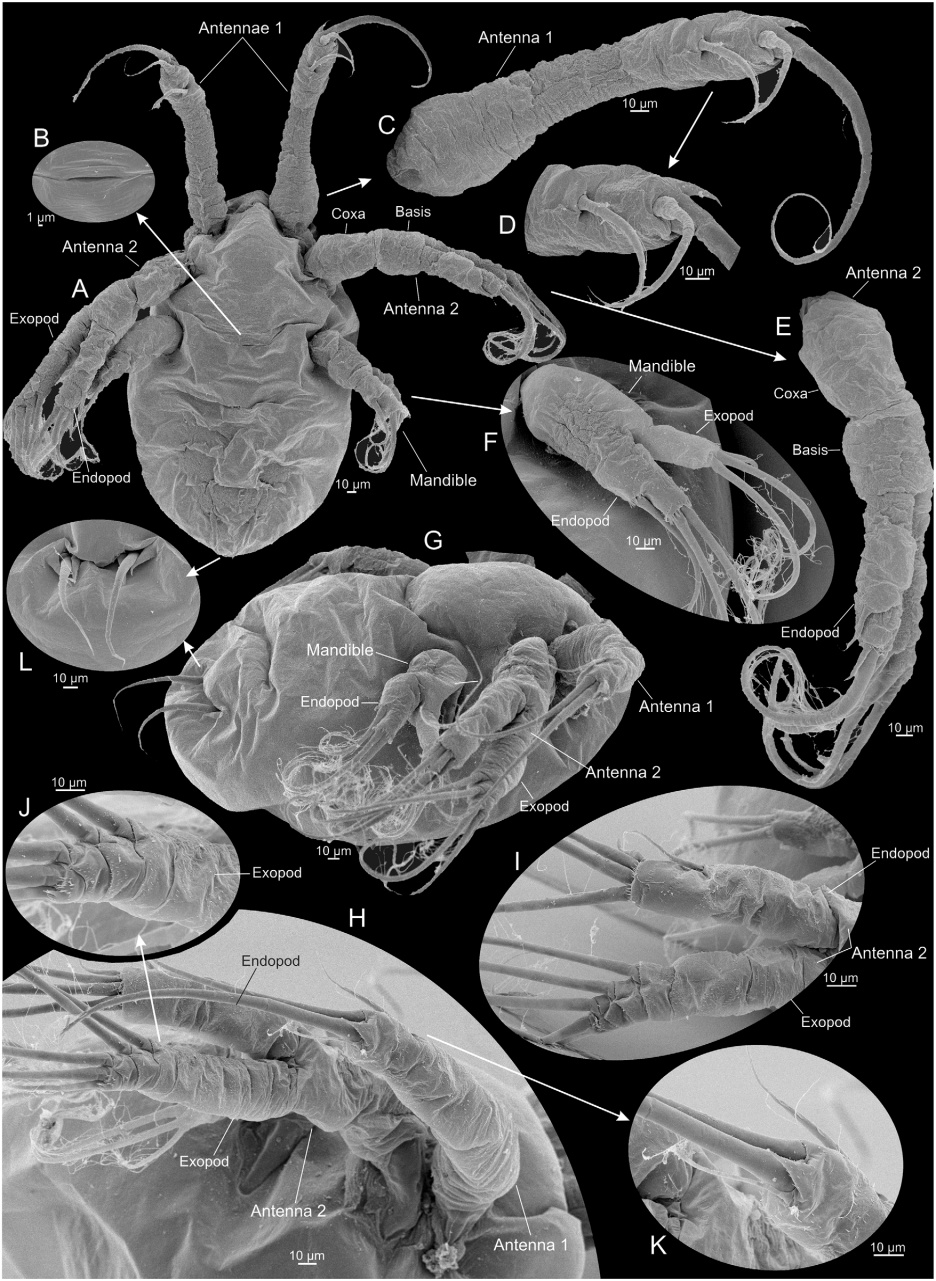
Nauplius 3 of *Thysanoessa raschii* (Euphausiacea), scanning electron microscopy (SEM). (A) Ventral view of whole specimen. (B) Incipient mouth opening (?). (C) Antenna 1, left side. (D) Tip of antenna 1. (E) Antenna 2, left side. (F) Mandible, left side. (G) Lateral view of whole specimen. (H) Antennae 1 and 2, left side, dorso-lateral view. (I) Antenna 2 endopod and exopod, left side. (J) Tip of antenna 2 exopod. (K) Tip of antennae 1.

**Fig 7:**
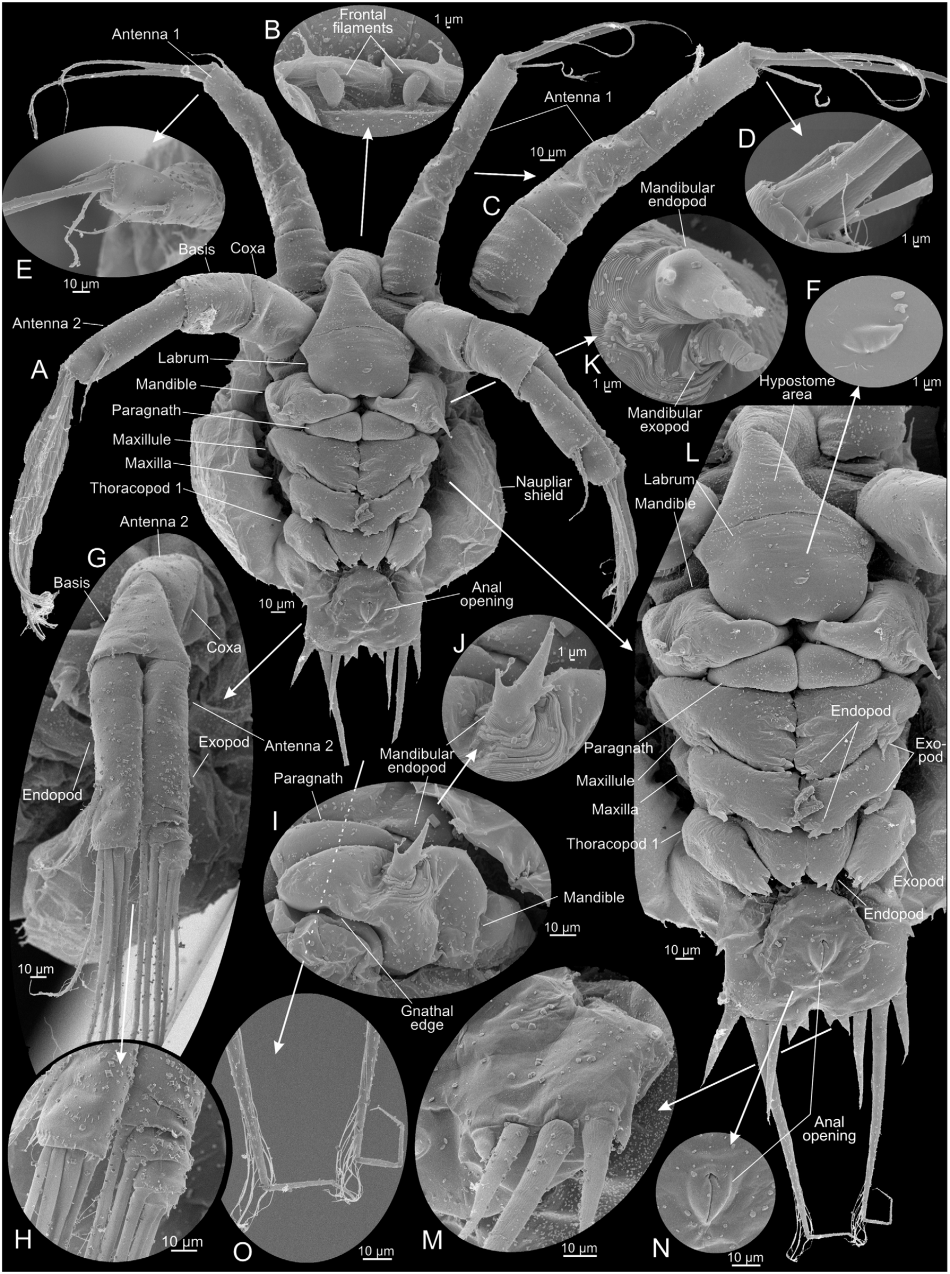
Metanauplius of *Thysanoessa raschii* (Euphausiacea), scanning electron microscopy (SEM). (A) Ventral view of whole specimen. (B) Frontal filaments. (C) Antenna 1, left side. (D) Tip of antenna 1, left side. (E) Tip of antenna 1, right side. (F) Cuticular structure of unknown significance on labrum. (G) Antenna 2, left side. (H) Tips of endopod and exopod of antenna 2. (I) Mandible and paragnath, right side, anterior view. (J) Rudimentary palp (endopodal part?) of mandible. (K) Rudimentary palp (endopod and exopod?) of mandible. (L) Ventral view of whole specimen with first and second antennae omitted. (M) Setation/spination of caudal lobe of left side. (N) Anal opening. (O) Setules of long caudal setae.

**Fig 8:**
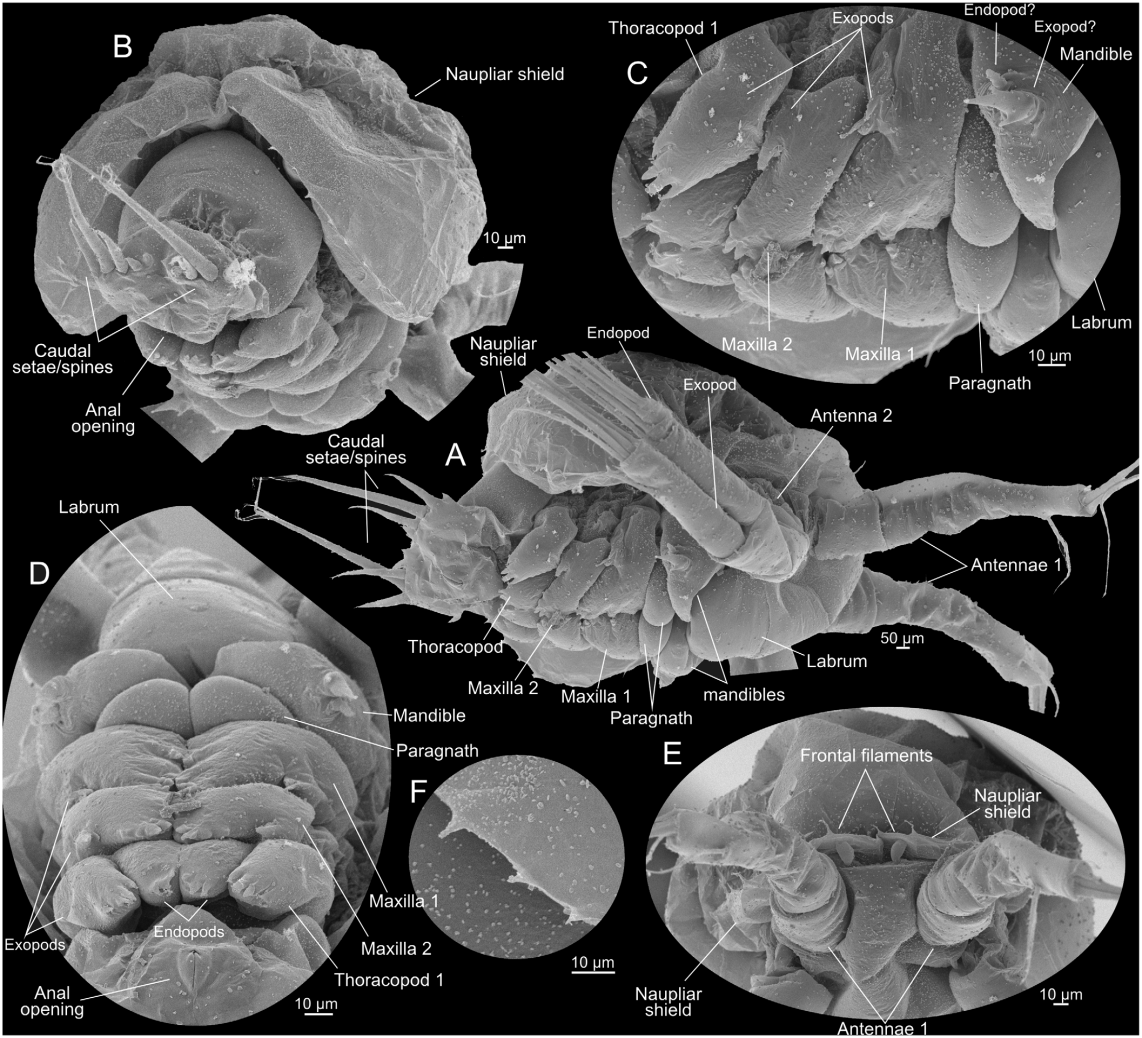
Metanauplius of *Thysanoessa raschii* (Euphausiacea), scanning electron microscopy (SEM). (A) Lateral view of whole specimen. (B) Caudal view of whole specimen. (C) Appendages (mandible, maxilla 1 and 2, thoracopod 1) of right side, lateral view. (D) Appendages (mandible, maxilla 1 and 2, thoracopod 1) of both sides, ventral view. (E) Frontal view showing antennae 1 and frontal filaments. (F) Marginal spines of posterior part of naupliar shield.

**Fig 9:**
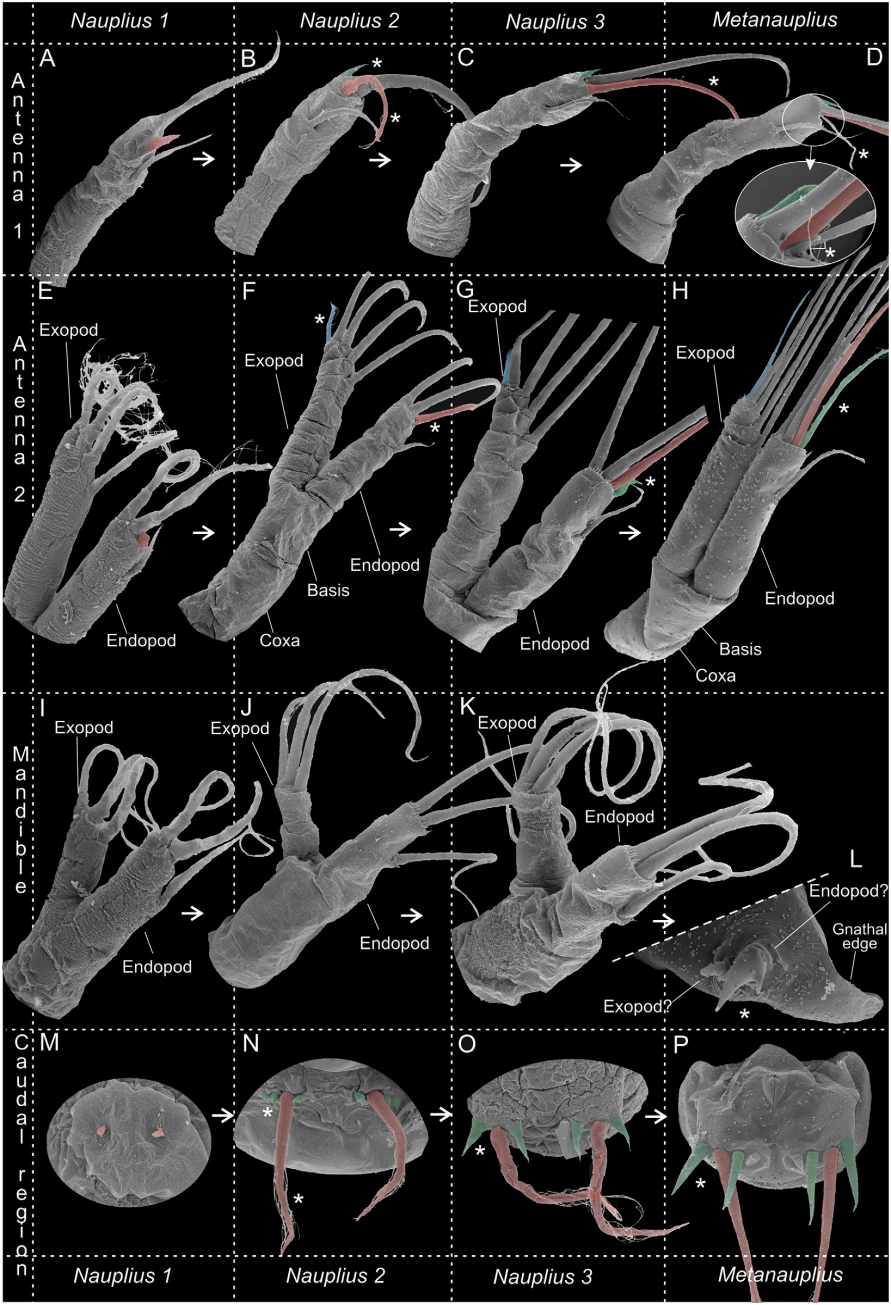
Overview of setation/spination of appendages and caudal region in nauplii 1–3 and metanauplius of *Thysanoessa raschii* (Euphausiacea). (A-D) Antenna 1. (E-H) Antenna 2. (I-L) Mandible. (M-P) Caudal region. * Indicates new structures or structures that are modified compared to the previous larval stage.

Descriptions based on Figs 3–8. Differences between stages summarized in Fig 9 and in Table 2.

**Table 2:** Setae and spines of nauplii 1–3 and metanauplius of *Thysanoessa raschii* (Malacostraca, Euphausiacea) with emphasis on structures useful for identification of different stages. This table corresponds to the overview of structures in Fig 9.

	Nauplius 1	Nauplius 2	Nauplius 3	Metanauplius
**Antenna 1**	*Terminally*: 1 long seta; 1 short spine. *Sub-terminally*: 1 medium-long seta (Fig 9A)	*Terminally*: 1 long seta; 1 medium-long seta*; 1 short spine*. *Sub-terminally*: 1 medium-long seta Fig 9B)	*Terminally*: 2 long setae*; 1 short spine. *Sub-terminally*: 1 medium-long seta (Fig 9C)	*Terminally*: 2 long setae; 1 short spine; 1 medium-long and slender seta*. *Sub-terminally*: 1 medium-long spine (Fig 9D)
**Antenna 2, endopod**	*Terminally*: 2 long setae; 1 small ‘hump’ (anlage of seta). *Sub-terminally*: 1 medium-long seta (Fig 9E)	*Terminally*: 2 long setae; 1 intermediate seta*. *Sub-terminally*: 1 medium-long seta (Fig 9F)	*Terminally*: 3 long setae*; 1 intermediate seta*. *Sub-terminally*: 1 medium-long seta (Fig 9G)	*Terminally*: 3 long setae; 1 intermediate seta. *Sub-terminally*: 1 medium-long seta (Fig 9H)
**Antenna 2, exopod**	4 long setae arranged ‘step-like’ (Fig 9E)	4 long setae arranged ‘step-like’; 1 intermediate seta terminally* (Fig 9F)	4 long setae arranged ‘step-like’; 1 intermediate seta terminally (Fig 9G)	5 long setae arranged ‘step-like’* (Fig 9H)
**Mandible, endopod**	*Terminally*: 2 long setae *Sub-terminally*: 1 long seta (Fig 9I)	*Terminally*: 2 long setae *Sub-terminally*: 1 long seta (Fig 9J)	*Terminally*: 2 long setae *Sub-terminally*: 1 long seta (Fig 9K)	Reduced to small, spinose structure* (Fig 9L)
**Mandible, exopod**	3 long setae terminally arranged weakly ‘step-like’ (Fig 9I)	3 long setae terminally (Fig 9J)	3 long setae terminally (Fig 9K)	Reduced to small, slender, ‘articulated’ structure* (Fig 9L)
**Caudal setae**	Absent or with small setae anlagen (Fig 9M)	Long with setules along distal half* (Fig 9N)	Long with setules along distal half (Fig 9O)	Long with setules along distal half (Fig 9P)
**Caudal spines** (articulated to hind body in metanauplius and could therefore also be termed ‘setae’)	Absent (Fig 9M)	Each long caudal seta with 3 small associated spines* (Fig 9N)	Each long caudal seta with 2 large associated spines* (Fig 9O)	Each long caudal seta with 2 large and 1 small associated spines* (Fig 9P)

* Indicates new structures or structures that are modified compared to the previous larval stage

#### Nauplius 1 (Figs 3A, 4, 9A, 9E, 9I and 9M)

The mean length of nauplius 1 is 0.45 mm with a large variation (0.37–0.51 mm); the mean width is 0.27 mm, also with a large variation (0.20–0.33) (Table 1, Fig 2).

The body is egg-shaped, about 1.5 times longer than wide (Figs 3A, 4B and 4C). Three pairs of naupliar appendages (antenna 1, 2, and mandible) are present ventrally on the body, concentrated in the anterior half (Figs 3A, 4B and 4C). All appendages extend somewhat latero-ventrally from the main body. The uniramous first antennae are about 2/3 as long as the body (without setation), curved slightly posteriorly, tubular, with distal setation consisting terminally of one setule-bearing long seta, one small spine, and one seta of intermediate length positioned at some distance from the tip (Figs 4D, 4E and 9A); some superficial subdivision of the surface in an uneven sclerotic arrangement can be seen, mostly in the proximal half where rows of large, quadrangular sclerites are present (Fig 4D). The biramous second antennae are about 4/5 as long as the body (without setation) and curved slightly posteriorly (3A, 4B-C). The protopod and the endopod are of about the same length, while the exopod is slightly longer. The protopod is weakly subdivided into a coxa and a basis (Fig 4C). The endopod bears three setule-bearing setae, two of which are placed distally and one at some distance from the tip; between the latter and the two distal setae is a small anlage of a fourth seta (Figs 4F and 9E, shaded red). The exopod bears four long, setule-bearing setae arranged weakly step-like along the medio-distal third of the ramus (4F, 9E). Neither the endopod nor the exopod is clearly segmented, but the dorsal surface of the exopod bears some plate-like scales (Fig 4D and 4F). The biramous mandibles are about 1/3 as long as the body (without setation) (Figs 3A and 4C). The protopod, the endopod, and the exopod are all of about the same length. The endopod bears three setulate setae, two of which are distal and one at some distance from the tip (Figs 4G and 9I); between the latter and the two distal setae is a small gap with what looks like an anlage of a fourth seta, but no such developed seta appears later during development. The exopod bears three large setulate setae distally arranged weakly step-like along the medio-distal third of the ramus (Figs 4G and 9I). There is much ornamentation externally, some of which is certainly a shrinking artifact of the critical point drying, but no consistent pattern could be identified. Some specimens show paired rudiments of the caudal setae terminally (Figs 4H and 9M). The descriptions of the following stages focus on those parts of the morphology where they deviate from the previous stage.

#### Nauplius 2 (Figs 3B, 5, 9B, 9F, 9J and 9N)

The mean length of nauplius 2 are 0.46 mm with a large variation (0.40–0.52 mm); the mean width is 0.26 mm also with a large variation (0.22–0.32) (Table 1, Fig 2).

The body of nauplius 2 is of approximately the same size as N1 but it has as more ellipsoidal shape (Figs 3B and 5A). Ventrally between the mandibles a small, slit-like opening has appeared, which is probably the incipient mouth opening (Fig 5C). The first antennae now bear four setae/spines distally (Figs 5A, 5D and 9B). Two of these are retained from N1, the spine from N1 has evolved into a true seta (colored red in Fig 9B), and a new spine has appeared anteriorly (shaded green in Fig 9B). The stem of the second antenna is now more clearly subdivided into a coxa and basis (Figs 5A, 5E and 9F). The endopod now bears four setae, in contrast to three in N1; the new seta (colored red in Fig 9F) is placed distally on the limb and is shorter than the two other distal setae. The exopod now bears five setae, in contrast to four in N1; the new seta is placed distally on the limb and is shorter than the four other setae (colored blue in Fig 9F). The dorsal side of the exopod is now more clearly subdivided into a row of 8–9 sclerites (Figs 5E and 9F). The mandibles of N2 are similar to those of N1 with respect to seta number (three on each ramus) (Figs 5F and 9J). The exopod is oriented more dorsally and bent away from the main axis of the limb, and the distal setation is arranged slightly less step-like than in N1 being more concentrated terminally (Fig 9J). A pair of large setulate caudal setae (Figs 5H, 5I and 9N, colored red in) has appeared on the body posteriorly on each side accompanied by small spines, two on the outer side of each of the caudal setae, one on the inner side (colored green in Fig 9N).

#### Nauplius 3 (Figs 3C, 6, 9C, 9G, 9K and 9O)

The mean length of nauplius 3 is 0.47 mm with a large variation (0.44–0.50 mm); the mean width is 0.27 mm also with a large variation (0.24–0.30) (Table 1, Fig 2).

The body of nauplius 3 is of approximately the same size as N1 and N2 but has an even more ellipsoidal shape than N2 (Figs 3C and 6A). The first antenna has the same distal setation as in N2 (Figs 6C, 6D and 9C). The second antenna endopod has four setae distally instead of the three seen in N2. The newly added setae is smaller than the remaining three (shaded green in Fig 9G) whereas one of the three setae seen already in N2 has grown considerably in size (shaded red in Fig 9G). The setation and general morphology of the mandibles are the same as in N2 (Figs 6F, 9J and 9K). Two of the spines flanking each caudal setae are considerably larger than in N2, but the third, outer one is unchanged (Figs 6L, 9N and 9O).

#### Metanauplius (Figs 3D, 7, 8, 9D, 9H, 9L and 9P)

The mean length of the metanauplius is 0.50 mm with a large variation (0.40–0.66 mm); the mean width is 0.33 mm, also with a large variation (0.23–0.49) (Table 1, Fig 2).

The metanauplius is morphologically very different from the previous stages and is therefore described in more detail. On average, the metanauplius is not much larger than N3, but there is a much larger size variation, both in length and width, among the examined specimens. A carapace fold (or naupliar shield) is present and is most pronounced anteriorly and posteriorly (Figs 7A, 8A, 8B and 8E). The anterior part of the fold extends approximately from the mandible and further anteriorly where it overhangs the proximal parts of the first and second antennae. At the rim of the anterior and posterior parts of the carapace fold are rows of small setae mostly arranged in an alternating pattern ‘small’, ‘large’, ‘small’, and so forth (pattern not shown on Figs). The posterior part of the carapace fold is wider than the anterior part and forms the widest point of the larva (Figs 3D and 7A). Posteriorly, the fold overhangs a small part of the ‘hind body’ and seems to be attached to the main body in the region of the anlage of maxilla 2 and/or thoracopod 1. At the rim of the posterior part of the fold are rows of small setae of slightly varying size; approximately 11–12 on each side. A pair of small filaments is present frontally under the anterior carapace fold between the first antennae (Figs 7B and 8E). A large U-shaped labrum is present being separated from a more anterior hypostome area by a suture (Fig 7L).

The first antennae are subdivided into a short proximal segment and a larger distal segment, which again is subdivided in 2–3 weakly defined portions (not examined further) (Figs 7A, 7C, 8A and 9D). The distal setation of the first antennae is almost like that of N3, except for the presence of an additional intermediate length seta and a small spine (Figs 7C, 7D and 9D). The second antennae differ from those of N3 in several aspects; the protopod is more clearly subdivided into a coxa and basis (Figs 7A, 7G and 9H) and the distal seta on the endopod, which appeared in N3, has become significantly longer (colored green in Fig 9H). Furthermore, the exopod still bears five setae distally but they appear more slender and more placed more distally on the ramus (Figs 7G, 8A and 9H) compared to N3 (Fig 9G). The weak subdivision of the exopod into dorsal sclerites seen in N2 and N3 is now lost and the ramus is smooth and undivided except in the distal region where it is subdivided into 3–4 segments corresponding to the setal arrangement. The endopod is unsegmented.

The mandible has undergone significant modification. It consists only of a large, swollen, blade-like structure, which is the coxa or a fusion product of the coxa and basis (see below), which medially extends into a lobe which will become the future gnathal edge (Figs 7L, 7I, 8C and 9L). At the ventro-lateral edge of the coxa (possibly combined with basis) is a small rudiment which certainly is the remains of the mandibular palp (endopod and exopod and possibly basis) (Figs 7I–7K, 8C and 9L). The rudiment is in two parts, median and lateral. The median part terminates in three spines, a large central one with a smaller one on each side; due to the median position of this part of the rudiment, it most likely corresponds to the endopod. The three spines are possibly the rudiments of the three long terminal setae of the mandibular endopod in the previous naupliar sequence. The lateral part is a small, ‘segmented’, tubular structure, which, due to its position, probably is the rudiment of the mandibular exopod from the naupliar sequence. If this interpretation is correct, it follows that the basipod has either been reduced or has fused with the coxa.

Posterior to the mandibles is a pair of undifferentiated paragnath buds (Figs 7L, 8C and 8D). Buds of three pairs of post-mandibular limbs are present: maxillae 1 and 2, and thoracopod 1 (Figs 7A, 7L, 8A, 8C and 8D). The first maxillae are limbs buds that are placed very closely together, with the median sides of the future limbs facing each other. An exopod anlage with two primordial spines is present laterally. Anlagen to what will become setae of the coxa, basis, and endopod are present medially, but the precise identity of these structures cannot be identified at present. The limb buds of the second maxillae are largely identical to those of the first maxillae, with the differences that the exopod anlagen consists of one spine only and the median side of the bud has fewer setae anlagen. The limb buds of thoracopods 1 each consists of a ventrally directed and well-defined endopod and exopod anlagen. The endopods are very close to each other. The endopod bears 2–3 setae anlagen, the exopod about four. The body terminates in a small, tubular ‘hind body’ which ends in a pair of short, dorso-ventrally flattened lobes each carrying five setae/spines (Figs 7L, 7M, 8A, 8B and 9P): one long setulate seta; two medium-long setae, one on each side of the long setae; and two small spines more medially. Ventrally at the ‘hind body’ is a slit-like anal opening (Fig 7N).

## Discussion

### Number of naupliar stages in broadcast-spawning euphausiaceans

The general aspects of the life cycle and development of euphausiaceans are relatively well-known (e.g., [36, 37]), but some details are still unclear, such as the precise number of stages in the early (naupliar) phase of the development of certain species. Because euphausiid species, such as the Antarctic krill *Euphausia superba* and the Northern krill *Meganyctiphanes norvegica* are of high ecological importance and link various trophic levels, this uncertainty regarding early development is unfortunate. Of the known 86 species of Euphausiacea, 61 are broadcast-spawners, shedding their eggs directly into the water, whereas 25 are sac-spawners, protecting the embryos in a membranous ovigerous sac attached to the last pair of thoracic legs [28, 38, 39]. Here we have studied the early (naupliar and metanaupliar) development of *Thysanoessa raschii*, sometimes termed Arctic krill, a broadcast-spawning euphausiid commonly found in various parts of the North Atlantic [29, 40–42].

**Fig 10:**
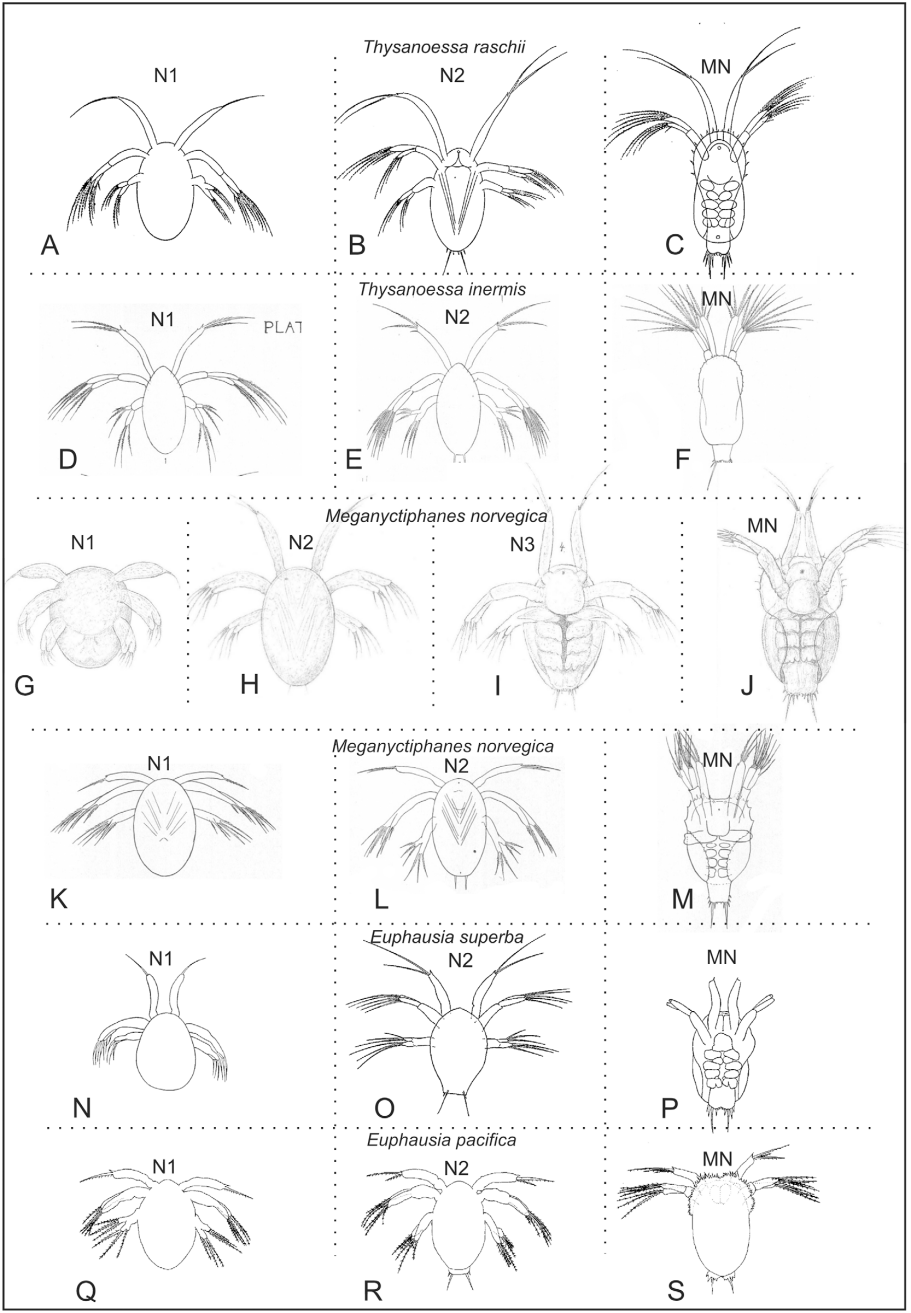
Overview of naupliar and metanaupliar stages of some of the species of Euphausiacea studied in most detail. (A-C) Two nauplii and one metanauplius of *Thysanoessa raschii* (from [43]). (D-F) Two nauplii and one metanauplius of *Thysanoessa inermis* (from [44]). (G-J) Three nauplii and one metanauplius of *Meganyctiphanes norvegica* (from [45]). (K-M) Two nauplii and one metanauplius of *Meganyctiphanes norvegica* (from [46]). (N-P) Two nauplii and one metanauplius of *Euphausia superba* (from [47]). (Q-S) Two nauplii and one metanauplius of *Euphausia pacifica* (from [48]).

We recognize three nauplius stages (N1-N3) and one metanauplius stage (MN) of *Thysanoessa raschii*, which is in contrast the two nauplius stages previously identified for this species [43], and also in contrast to most other broadcast-spawning euphausiaceans (see Table 3, Fig 10). With regard to the three naupliar stages of *T*. *raschii*, the most significant changes take place between nauplius 1 and 2, while the changes between nauplius 2 and 3 are more subtle (Table 2, Fig 9). Among the changes between nauplius 1 and 2 are: (1) a general change in body shape from more rounded plumb-shaped to more elongate (Fig 3A and 3B); (2) first antennae with one more spine and longer terminal seta (asterisks on Fig 9B); (3) second antennae with one additional seta terminally of both rami (asterisks on Fig 9F); and, most significantly, a pair of long caudal setae with small anlagen of flanking spines (asterisks on Fig 9N). The morphological changes between nauplius 2 and 3 are much smaller but involve at least two aspects: (1) the appearance an additional seta at the tip of the antennal endopod (asterisk on Fig 9G); and (2) larger size of the caudal spines flanking the large setae (asterisk on Fig 9O). Assuming that the identified nauplius stages actually belong to the same species (*Thysanoessa raschii*, see Materials and Methods), we consider that the three nauplius stages represent three different instars separated by molts. Certainly the large changes between N1 and N2 can only be explained by a molt, and probably a molt between N2 and N3 is necessary to explain the increased size of the caudal spines and the additional seta of the antennal endopod seen in N3. However, in principle, the occurrence of molting between stages (which then are ‘instars’) can only be identified with certainty by detailed studies of live material, ideally by following isolated individuals through their development, e.g., by examining the exuvia left behind. The original work on the development of *T*. *raschii* was indeed partly based on cultured material [43], so it is not easy to understand why a different number of nauplius stages was then recognized. Since the morphological differences between nauplius 2 and 3 found by us are really small and only concerns the caudal spination and one additional antennal endopod setae, on which was provided no details in [43], it is possible that one stage (N2) was not recognized in that work.

**Table 3:** Summary of naupliar and metanaupliar development in broadcast-spawning species of euphausiaceans. Only information from papers providing original contributions has been included. Some of the species included in the table are illustrated in Fig 10.

Species	N1	N2	N3	MN	Comparison with *T*. *raschii* with respect to key characters (caudal spination and antennal setation)	Literature source
***Thysanoessa raschii***	X	X	X	X		This study
***Thysanoessa raschii***	X	X		X	‘Nauplius 2’ in [43] is, based on caudal spination, most similar to nauplius 3 of this study. Antennal setation was not studied.	[43]
***Thysanoessa inermis***	X	X		X	Naupliar stages were not studied in detail regarding caudal spination and antennal setation.	[44]
***Meganyctiphanes norvegica***	X	X	X	X	Three nauplii is depicted in [45]: 1) A ‘recently hatched nauplius’, a ‘more advanced nauplius’; and a ‘last naupliar stage’. The text of [45] is not conclusive on this matter. Based on the degree of development of the caudal setae and spines, the ‘more advanced’ Nauplius in [45] is equivalent to nauplius and the ‘last naupliar stage’ to nauplius 3 of *T*. *raschii*.	[45]
***Meganyctiphanes norvegica***	X	X		X	Naupliar stages were not studied in detail regarding caudal spination and antennal setation. Based on live material it was stated that the first nauplius becomes the second nauplius and the second nauplius was said to change into the metanauplius.	[46]
***Meganyctiphanes norvegica***	X	X		X	Two different forms of nauplius 2 were reported, one with two caudal setae, each with two small caudal spines associated, and another in which these spines are larger.	[49]
***Euphausia superba***	X	X		X	Nauplius 2 in [47] is not precisely equivalent to either N2 or N3 *of T*. *raschii*. The caudal setae each have only one smaller associated spine (laterally), in contrast to 2–3 in *T*. *raschii*, and the setation of various appendages is in part different. The setation of the antennal endopod (3 long setae and a short) is similar to that of N2 of *T*. *raschii*. The exopod with its 6 setae has no equivalent in *T*. *raschii*. Note that the endopod and the exopod of A2 were reversed in [47].	[47, 52]
***Euphausia superba***	X			X	Based on live material, the first molt was found to be between the ‘nauplius stage’ and the metanauplius.	[53]
***Euphausia gibboides***	X	X		X	Nauplius 2 is closest to nauplius 2 of *T*. *raschii* in having small outer spines of the caudal region and the antennal endopods with 3 terminal and 1 subterminal setae.	[50])
***Euphausia nana***	X	X		X	Nauplius 2 is closest to nauplius 2 of *T*. *raschii* in having small outer spines of the caudal region and the antennal endopods with 3 terminal and 1 subterminal setae.	[51]
***Euphausia pacifica***	X	X		X	Nauplius 2 is closest to nauplius 2 of *T*. *raschii* in having small outer spines of the caudal region and the antennal endopods with 3 terminal and 1 subterminal setae.	[48]

Most studies of euphausiid development state that the early development of broadcast-spawning species consists of two naupliar stages, one without caudal setae, and one with caudal setae. However, the present work suggests that it is more complicated. With one exception [45], the present study is the first report of three naupliar stages for any species of Euphausiacea. All other studies, including, as mentioned, the previous treatment of *T*. *raschii* [43], have identified only two naupliar stages (Table 3, Fig 10). Without detailed re-examination of the naupliar development of these other species, with particular attention to caudal spination and setal pattern of various limbs, which have proven crucial for stage identification in *T*. *raschii*, it is not possible to conclude with certainty whether a nauplius stage has been missed for some of these other species, as may have been the case in the previous treatment of *T*. *raschii* [43]. In the following, we discuss the number of naupliar stages in those species of the Euphausiacea for which most details are known.

In a work on the development of *Thysanoessa inermis*, two naupliar stages were depicted and described very briefly (Fig 10D–10F) [44]. No attention was paid to the detailed morphology of the caudal spines and the setation of the antennal endopod, which have proved relevant for distinguishing between N2 and N3 of *T*. *raschii*. Therefore, there is a possibility that a stage was overlooked for *T*. *inermis*. *Meganyctiphanes norvegica* is the only other euphausiid in which three naupliar stages have been described [45]; however, conflicting evidence exists. The development of *M*. *norvegica* was later re-studied [46], apparently on the basis of live material, and it was specifically stated that the second nauplius followed from the first nauplius and that the metanauplius followed from the second nauplius, thus yielding one fewer naupliar stage than had been found previously [45] (Fig 10K–10M). Caudal spines and antennal setation were not reported in detail in either of the two works. More information on the same species was later added [49]. Among other, two different forms of N2 were found, which differed from each other with respect to the size of the spines associated with the long caudal setae. The more developed one of these ‘N2’ (the one with long caudal spines) may be equivalent to N3 of *T*. *raschii* as recognized here, but the confirmation of this will require a restudy. In all cases when the development of *Euphasia* has been examined, two naupliar stages have been recognized [48, 50–52] (e.g., for *E*. *superba* and *E*. *pacifica* in Fig 10N–10S). In most such cases, the setation patterns of the naupliar appendages have been examined in detail. This is in contrast to most of the species mentioned above. It thus seems plausible that only two naupliar instars are present in species of *Euphasia*. With respect to the characters that have proved important for distinguishing between N2 and N3 of *T*. *raschii* (size of caudal spines and setation of the antennal endopod), N2 of the various species of *Euphasia* looks most like N2 of *T*. *raschii*.

A general question concerns whether the ‘stages’ recognized here for the naupliar development of *T*. *raschii* corresponds to instars separated by molts. The approach used, which is a widespread method, has been to examine the morphology of a large number of individuals and to consider whether the changes in morphology between stages are too large to be explained by a molt. Certainly the multiple differences between N1 and N2 in *Thysanoessa raschii* are too large to have occurred without a molt, but what about the more minute changes between N2 and N3? However, while the growth of the cuticular caudal spines (see Fig 9N and 9O) might be explainable as simple cuticular expansion, even though we lack evidence that such can happen, the addition of an extra seta (see Fig 9F and 9G) is difficult to explain this way. We therefore consider that the three naupliar stages of *T*. *raschii* recognized here correspond to three separate instars. A similar question was considered earlier by other researchers for *Euphausia superba*, with a completely different conclusion [53]. According to that study, all the naupliar developmental stages from hatching to the metanauplius occurred without molting, which, if true, means that all the naupliar stages for *Euphasia superba* belongs to one very ‘inclusive’ instar, and that all the observed morphological changes, including the appearance of long caudal setae, would have occurred by cuticular expansion. In this regard, [53] is in conflict with all other thorough studies on the development of euphausiaceans, whereby always at least two naupliar stages have been identified and in some cases specifically stated to be separated by a molt (e.g., [46]). However, [53] is not easy to dismiss, since the methodology seems properly set up to detect the presence/absence of molting in *E*. *superba*, for example by basing the work on cultures of live material. The whole issue is of wider importance and needs general attention. Intermolt cuticular modifications as a part of the molting cycle are well-known for arthropods [54], but in our view it is doubtful that intermolt additions of external surface structures, such as the caudal spines and limb setation as described here for *T*. *raschii*, can take place.

### The nauplius stage in malacostracan evolution

The presence of a naupliar phase of the development in most euphausiaceans is important in a broader evolutionary context because it is widely accepted that a naupliar phase was part of the earliest development of Crustacea (= Pancrustacea or Tetraconata) [5, 6, 26, 55, 56]. This idea is built primarily on two types of argument: (1) the taxonomically widespread occurrence of nauplii in many very different crustacean taxa (e.g., branchiopods, cirripedes, and copepods, as well as certain malacostracans), and (2) the fundamental similarity between the nauplii of these taxa (e.g., with only three pairs of appendages: first antenna, second antennae, and mandibles). For a long time it was accepted that the occurrence of a naupliar phase in the early development of two malacostracan taxa, euphausiaceans and dendrobranchiate shrimps, reflects the ancestral condition for Malacostraca (e.g., [23–25]) from which other types of development in Malacostraca had been derived. This idea was challenged by [26] who found evidence that the free-living nauplius larvae of malacostracans having evolved secondarily from ontogenies without a free-living nauplius (see details below), an idea that had been discussed earlier [27] and was later explored further [57, 58]

Here, we re-address this question based on the new data obtained for *Thysanoessa raschii* (Euphausiacea) with a comparison to the early (naupliar) development of dendrobranchiate decapods and some non-malacostracan crustaceans.

The arguments in [26] for secondary evolution of free-living nauplii within Malacostraca were a combination of parsimony and morphology. As remarked in [26], despite the uncertain malacostracan phylogeny, there is consensus that both euphausiaceans and dendrobranchiate decapods should be placed within the Malacostraca, perhaps even as sister taxa (e.g., [59]), with several other malacostracan taxa having branched off earlier than these two. Depending on the precise branching pattern within the Malacostraca, this would require an independent loss of the free-living nauplius a number of times [26]. The other main argument in [26] was a striking similarity in the morphology of certain early developing stages (‘egg-nauplii’) in those Malacostraca that lack free-living nauplii. Many taxa (e.g., Leptostraca, Stomatopoda, Thermosbaenacea, and some peracarids) share a particular resemblance in the post-naupliar body region, including the presence of a growth zone with a fixed number of ectoteloblasts (19) and a ventrally flexed caudal papilla. If these similarities are homologous, they can be argued to have been present in the ground pattern of the Malacostraca [26]. However, if these similarities are considered independently, the support for ‘egg-nauplii’ ancestrally in Malacostraca weakens. Concerning the fixed number of ectoteloblasts (19) and mesoteloblasts (8), then such a pattern is also found in free-living nauplii of Euphausiacea [26] and therefore not relevant for the question of whether ‘egg-nauplii’ or free-living nauplii where present ancestrally in Malacostraca. Concerning the ventrally flexed caudal papilla this type of embryonic flexing seems to be an obvious way to accommodate to the limited space available for growth within an egg-shell, and is therefore prone to convergence; ventral flexing is seen also in the non-related Cephalocarida [17, 60]. It must be admitted that evolutionary questions such as whether free nauplii were present or not ancestrally in early development in Malacostraca, are very difficult to answer with certainty. The most compelling evidence would be fossil malacostracan nauplii belonging to early off-splits of malacostracans, e.g., Phyllocarida. Ancient fossil nauplii from the Cambrian and the Devonian have been found for a number of non-malacostracan taxa, including cirripedes and branchiopods, and for some taxa of uncertain affinity [1, 19, 20, 61–63], but none of them have been assigned to Malacostraca. So the question will have to be addressed by more circumstantial arguments based on (1) parsimony (e.g., [26]), (2) morphological analysis of development within Malacostraca (e.g., [26, 57]), and (3) morphological comparison with taxa outside the Malacostraca (outgroup comparison).

(1) and (2) were explored in [26] (addressed above), so here we examine the third type of argument (outgroup comparison), by means of a comparison with the early naupliar development of some non-malacostracan crustaceans. If detailed similarities can be established between, on one side, the naupliar phase of euphausiaceans (krill) and dendrobranchiate shrimps and, on the other side, the naupliar phase of non-malacostracans, including some of the amazingly preserved and important ‘Orsten’ taxa (e.g., *Rehbachiella kinnekullensis*), then this will be an argument in favour of the primitive status of the free-living larvae within the Malacostraca.

Indeed there are numerous both general and detailed similarities between the development of non-malacostracan taxa such as branchiopods (incl. *Rehbachiella*) and euphausiaceans and dendrobranchiate shrimps.

**Fig 11:**
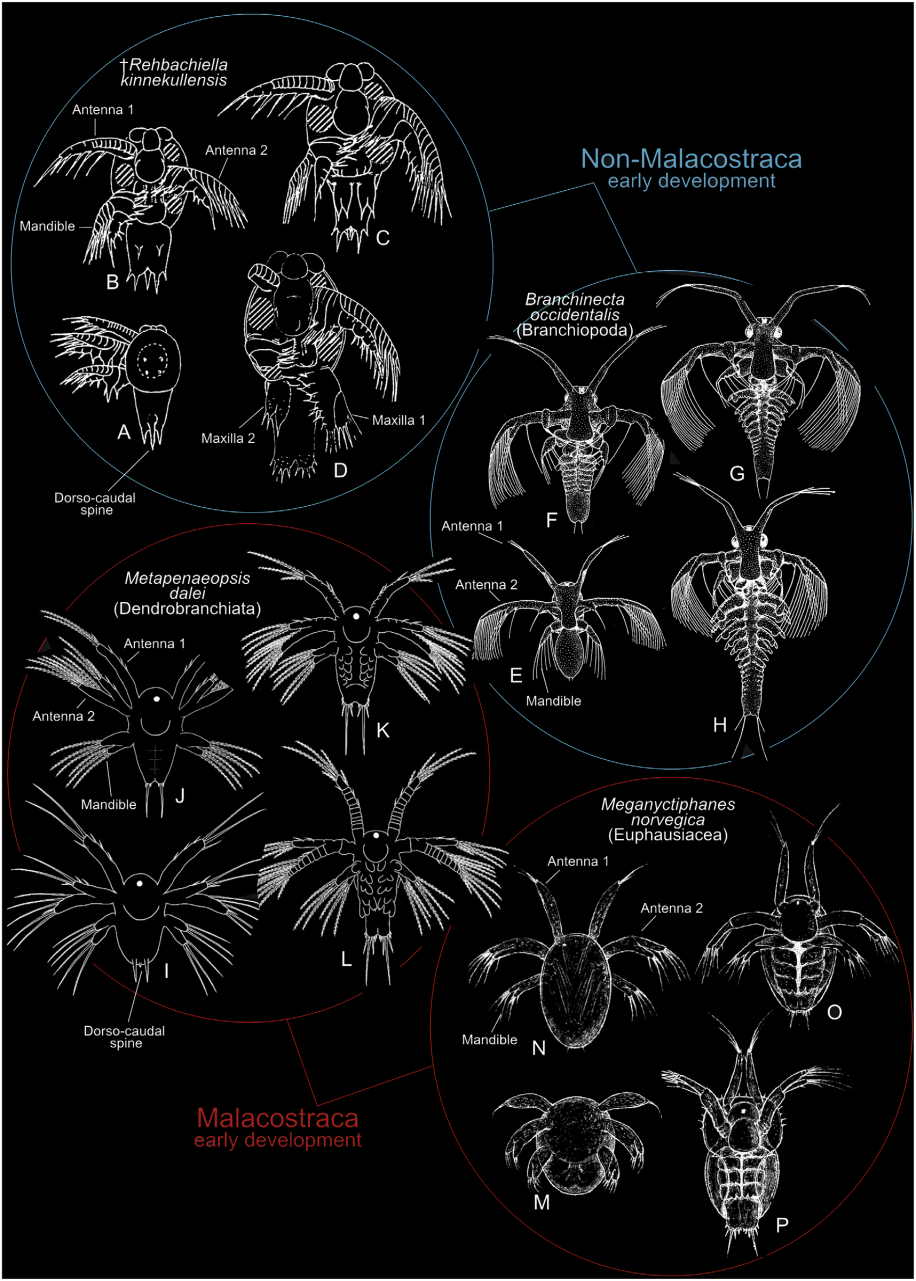
Early (= naupliar type) development of two malacostracan taxa and two non-malacostracan. (A-D) Four naupliar stages of the Cambrian ‘Orsten’ crustacean *Rehbachiella kinnekullensis* (from [20]). (E-H) Four naupliar type stages of *Branchinecta occidentalis* (Anostraca) (from [75]). (I-L) Four (out of six) naupliar stages of *Metapenaeopsis dalei* (Dendrobranchiata Decapoda) (from [73]).

These similarities include:

1) An overall similarity between the nauplii of all taxa regarding the number and general morphology of the naupliar appendages: uniramous first antennae, biramous second antennae, biramous mandibles (except branchiopods) (see general morphology of nauplii of some taxa in Fig 11).

**Fig 12:**
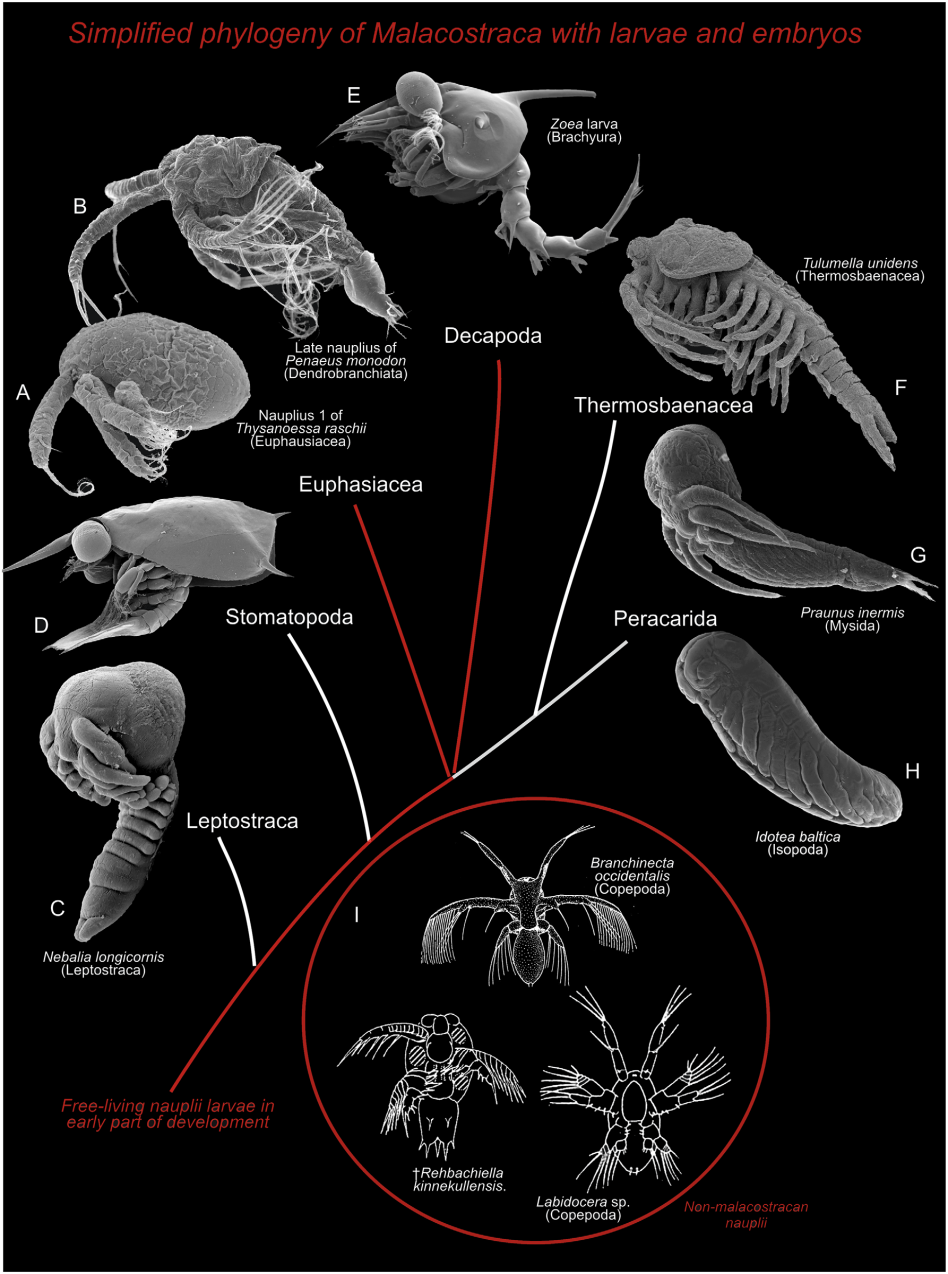
Evolution of naupliar development mapped on a simplified phylogeny of the Malacostraca based on several papers providing partly conflicting phylogenetic results [76, 77]. The figure summarizes the idea supported in this paper, that the naupliar type of development seen in two malacostracan taxa, Euphausiacea and dendrobranchiate decapods (A and B), in essence is primitive for malacostracans and retained, but modified, from earlier in evolution (red line). C-H show other examples of the variation in developmental type in Malacostraca, both of taxa with free-living larvae and direct developers. (C) Early embryonic-like stage of *Nebalia longicornis* (Leptostraca) (from [78]). (D) Antizoea larva (Stomatopoda). (E) Zoea larva (Brachyura). (F) Late development stage of *Tulumella unidens* (Thermosbaenacea) (from [14]). (G) *Praunus inermis* (Mysida) (from [79]). (H) *Idotea baltica* (Isopoda) (material collected in Denmark by JO). (I) Three examples of non-malacostracan taxa with free living nauplii early in their development (Branchiopoda, Copepoda, and the Cambrian *Rehbachiella*).

Detailed similarities:

2) In the earliest stages of *Thysanoessa raschii* (Euphausiacea) the cuticle of some of the naupliar limbs are not arranged clearly into well-defined segments, but rather into a loosely defined pattern of sclerites. This is especially characteristic for the first antennae (e.g., Figs 5D, 6C, 9A–9C), but also other naupliar appendages. This is not unique for euphausiid larvae but is also seen in dendrobranchiate decapods (see Fig 12) and many non-malacostracan taxa such as branchiopods [64], and ‘Orsten’ crustaceans such as *Rehbachiella kinnekullensis* [20].
3) The dorsal side of the antennal exopod of *Thysanoessa raschii* of the early naupliar stages is arranged into a row of cuticular ‘half rings’; approximately seven in nauplius 2, which is the stage where the ‘half rings’ are most pronounced (Fig 5E). These ‘half rings’ could be rudiments of an earlier more pronounced segmentation. A variation over this type of antennal exopodal morphology is common for Crustacea, also for the mandibular exopod, and seen in many taxa (e.g., branchiopods, mystacocarids, cephalocarids, ‘Orsten’ crustaceans [20, 64–67], and dendrobranchiate decapods (Fig 12), where rows of complete ringlets are seen, each of which correspond to one seta
4) The setation of the naupliar limbs are in general arranged the same way in *Thysanoessa raschii* and non-malacostracan nauplii: Antenna 1 with setae concentrated at the tip; endopods of antenna 2 and mandible with setae mostly at the tip, exopods with row of setae (one for each ‘ringlet’) along inner margin.
5) The caudal spination/setation in *Thysanoessa raschii* and in other euphausiaceans starts in nauplius 2 with a pair of long setulated setae, which in the following stages become flanked by small spines that develops into distinct setae in the metanauplius. The caudal setation/spination basically starts in the same way in dendrobranchiate decapods and in non-malacostracan taxa such as the Anostraca, Copepoda, and *Rehbachiella kinnekullensis* (e.g., see some taxa in Fig 11). The further development of the caudal setation/spination is very different in various taxa, but this is beyond the scope to explore here.
6) The naupliar shield in the metanauplius of *Thysanoessa raschii* (Fig 8A and 8B) and other euphausiaceans is a posterior extension of the head region (see above), not fundamentally different from what is seen in dendrobranchiate decapods and in many non-malacostracan crustaceans (see [68]).
7) The metanauplius of *Thysanoessa raschii* has a pair of frontal filaments (Figs 7B and 8E) very similar to those seen in the larvae of some non-malacostracans, such as those of *Lynceus* (Branchiopoda) and other branchiopods, which, at least in branchiopods, are external extensions of an internal ‘frontal filament organ’ [9, 15, 69]. Resembling structures, sometimes termed ‘Organs of Bellonci’, are present in other crustaceans such as barnacle nauplii and larval and adult Remipedia (see [21, 70] and summary in [9]).
8) No dorso-caudal spine has been found in any of the examined early stages of *Thysanoessa raschii*, but a rudiment is present in nauplius 1 of *Metapenaeopsis dalei* (see Fig 11I) representing Dendrobranchiata, the other malacostracan group with free-living nauplii. A ‘dorso-caudal spine’, a characteristic spine-like protrusion dorsally at the hind body during early development in many non-malacostracans such as mystacocarids, cirripedes, and in ‘Orsten’ larvae such as *Rehbachiella kinnekullensis* and *Bredocaris admirabilis* (e.g., [19, 20, 71, 72]), and its presence in *Metapenaeopsis dalei* (Dendrobranchiata) can be explained as a plesiomorphy (rudiment), and its absence in Euphausiacea as secondary loss.
9) A general similarity between euphausiaceans and dendrobranchiates on one hand and many non-malacostracans on the other, is seen in the gradual development of the naupliar part of their ontogeny (later part of development not treated here). The six nauplii known for *Metapenaeopsis dalei* (Dendrobranchiata) develops gradually from stage to stage with respect to limb addition/setation and caudal setae/spine addition [73]. Structures related to feeding (labrum, mandible) is clearly delayed, but this is also seen in some non-malacostracans such as branchiopods (e.g., [74]), though to a lesser degree, but not fundamentally different. The naupliar phase in Euphausiacea, as exemplified in this study of *Thysanoessa raschii*, is less gradual than that of dendrobranchiates and involves only three naupliar stages followed by a distinct jump in morphology between the naupliar phase and the metanauplius. However, the development of setation and segmentation of the naupliar appendages is still essentially gradual, as is the development of caudal setae/spines; a clear delay is seen in the development of structures relating to feeding as in dendrobranchiates. Hence, with respect to gradualness, the naupliar development of both Euphausiacea and Dendrobranchiata falls within the range of those non-malacostracans which start with a naupliar sequence (except for the delayed feeding structures) (see summary in [6]).

All these similarities between nauplii of euphausiaceans, dendrobranchiates and non-malacostracan nauplii are simplest to explain as homologous, which leads to suggesting that free-living nauplii within the Malacostraca have been retained from earlier in evolution, although modified in various ways; for example to a lecitotrophich lifestyle to a degree not seen in the development of non-malacostracan crustaceans, with the exception of the cave dwelling Remipedia, which seems to undergo an even more extended lecitotrophich development [21, 22].

If free-living nauplii in Euphausiacea and Dendrobranchata Decapoda indeed have been retained from earlier in evolution, as argued here, then it is true that such larvae must be considered lost a number of times within Malacostracan depending on the favored phylogeny. But when looking in detail at the various types of ‘direct development’ within various taxa, some of which have an ‘egg nauplius’ (see summary in [26]), then it is clear that ‘direct development’ of Malacostraca is an assemblage of very different developmental types, and furthermore, involves many different types of brooding by the mother animal. This is not the place to summarize all the developmental modes in detail, but a brief comparison shows that the gross morphology of embryos of, for example, *Nebalia* (Leptostraca), *Tulumella* (Thermosbaenacea), and *Idotea* (Isopoda) is rather different (see Fig 12). These differences do not add support to the notion of a common origin of direct development in these taxa.

Furthermore, brooding in these three malacostracan taxa are facilitated by very different parts of the body of the mother animal. In the case of leptostracans, brooding takes place ventrally between the mother’s thoracopods, which form a large basket holding the developing embryos, while brooding in thermosbaenaceans takes place dorsally under an enlarged part of the carapace, and that of isopods take place ventrally in a marsupium formed, probably, by modified thoracopodal epipods. A further example is in Pleocyemata (Stenopodidea, Caridea, and Reptantia), where developing embryos are kept attached to pleopods under the pleon of the female. It is, therefore, plausible that direct development has evolved in various malacostracan taxa independently, and this notion supported, in our view, by the fact that the brooding is facilitated by so many different parts of the mother body in different taxa. However, many details needs to be elucidated, and all evolutionary conclusions also depends on the phylogeny of the Malacostraca, concerning which there is still uncertainty (e.g., [59, 76]).

Returning to the question of the evolutionary status of the free-living nauplii of euphausiaceans and dendrobranchiates, a number of ‘embryonic’ characters were mentioned in [26] for the larvae of both taxa in support of their secondary origin within Malacostraca. Most of these relate to the non-feeding status of the larvae (fully formed gut absent, body with yolk cells, labrum absent, setae only at tips of limbs, mandibular gnathobase and antennal masticatory spines absent), and may as well be explained as adaptations to lecitothrophy, not necessarily indicating an embryonic origin. Early nauplii of other crustaceans such as some branchiopods, copepods, and cirripedes also go through a lecitothrophic phase during which they in some cases are expanded by yolk and their naupliar feeding appendages are inactive [74, 80–83]. Furthermore, in support of an eventual embryonic origin, it has been mentioned that the limbs are ‘not articulated’ [26]. However, as noted above, the indistinct and somewhat disorganized arrangement of the cuticle of the naupliar appendages is not confined to euphausiid and dendrobranchiate nauplii but is found in the early larvae of many other taxa as well. Most recently euphausiaceans and dendrobranchiates have been suggested to be sister taxa based on sequence similarities of the mitochondrial genome [59], which, if true, would leave the Decapoda paraphyletic. Such a putative sister-group relationship is interesting in the light of the shared naupliar type of development between these two malacostracan taxa, but does not challenge the above arguments in favour of an ancestral status of naupliar development for Malacostraca.
